# Crystal structure of *tert*-butyl­diphenyl­phosphine oxide

**DOI:** 10.1107/S2056989015008919

**Published:** 2015-05-13

**Authors:** George Agbeworvi, Zerihun Assefa, Richard E. Sykora, Jared D. Taylor

**Affiliations:** a 1601 E Market St., Department of Chemistry, North Carolina A & T State University, Greensboro, NC 27411, USA; b University of South Alabama, Department of Chemistry, Mobile, AL 36688-0002, USA

**Keywords:** crystal structure, phosphine oxide

## Abstract

In the structure of the title triorganophosphine oxide, C_16_H_19_OP, the P—O bond is 1.490 (1) Å. The P atom has a distorted tetrahedral geometry. The O atom inter­acts with both phenyl groups of a neighboring mol­ecule [C⋯O = 2.930 (3) and 2.928 (4) Å]. The C—O interaction directs an extended supramolecular arrangement along the *a*-axis.

## Related literature   

For the structures of related phosphine oxides Ph_3_P=O, EtPh_2_P=O and BuPh_2_P=O, see: Al-Farhan (1992 ▸), Orama & Koskinen (1994 ▸) and Caddy *et al.* (2007 ▸), respectively.
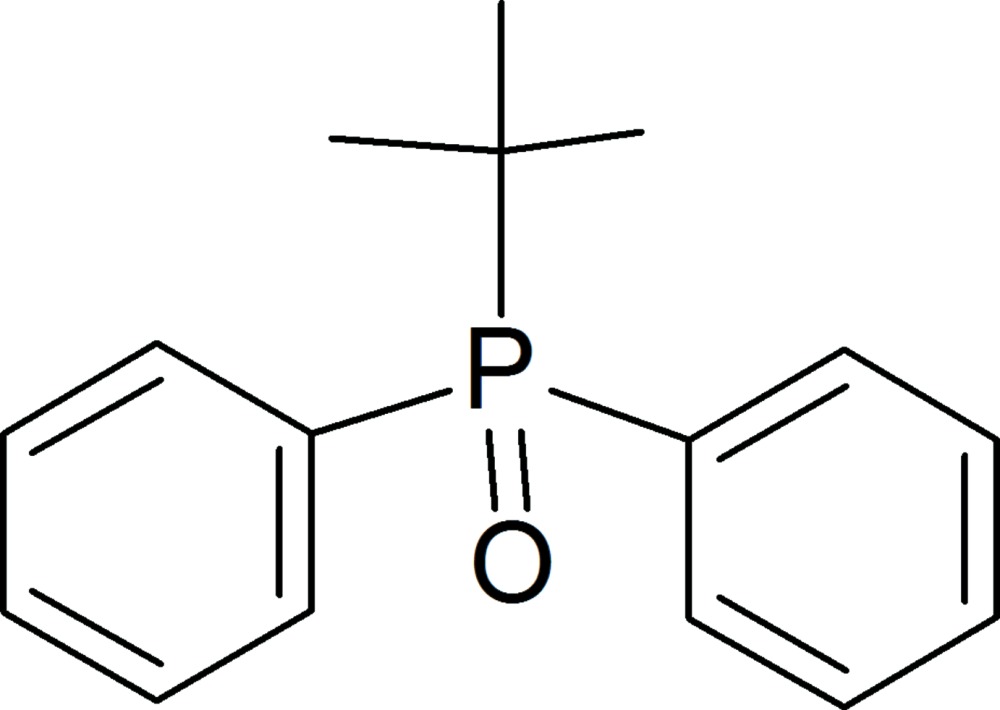


## Experimental   

### Crystal data  

C_16_H_19_OP
*M* *_r_* = 258.28
Monoclinic, 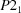

*a* = 6.3432 (6) Å
*b* = 9.5219 (9) Å
*c* = 12.4556 (15) Å
β = 101.665 (10)°
*V* = 736.78 (13) Å^3^
*Z* = 2
Mo *K*α radiation
μ = 0.17 mm^−1^
*T* = 293 K
0.4 × 0.15 × 0.04 mm

### Data collection  

Agilent Xcalibur, Eos diffractometer
Absorption correction: multi-scan (*CrysAlis PRO*; Agilent, 2014 ▸) *T*_min_ = 0.842, *T*_max_ = 1.000
11029 measured reflections
2713 independent reflections
2203 reflections with *I* > 2σ(*I*)
*R*_int_ = 0.049

### Refinement  

*R*[*F* ^2^ > 2σ(*F* ^2^)] = 0.044
*wR*(*F* ^2^) = 0.086
*S* = 1.05
2713 reflections
166 parameters
1 restraint
H-atom parameters constrained
Δρ_max_ = 0.18 e Å^−3^
Δρ_min_ = −0.24 e Å^−3^
Absolute structure: Flack (1983 ▸)
Absolute structure parameter: 0.19 (10)

### 

Data collection: *CrysAlis PRO* (Agilent, 2014 ▸); cell refinement: *CrysAlis PRO*; data reduction: *CrysAlis PRO*; program(s) used to solve structure: *SHELXS97* (Sheldrick, 2008 ▸); program(s) used to refine structure: *SHELXL97* (Sheldrick, 2008 ▸); molecular graphics: *OLEX2* (Dolomanov *et al.*, 2009 ▸); software used to prepare material for publication: *OLEX2* and *publCIF* (Westrip, 2010 ▸).

## Supplementary Material

Crystal structure: contains datablock(s) I, New_Global_Publ_Block. DOI: 10.1107/S2056989015008919/hg5439sup1.cif

Structure factors: contains datablock(s) I. DOI: 10.1107/S2056989015008919/hg5439Isup2.hkl

**Fig. 1.**
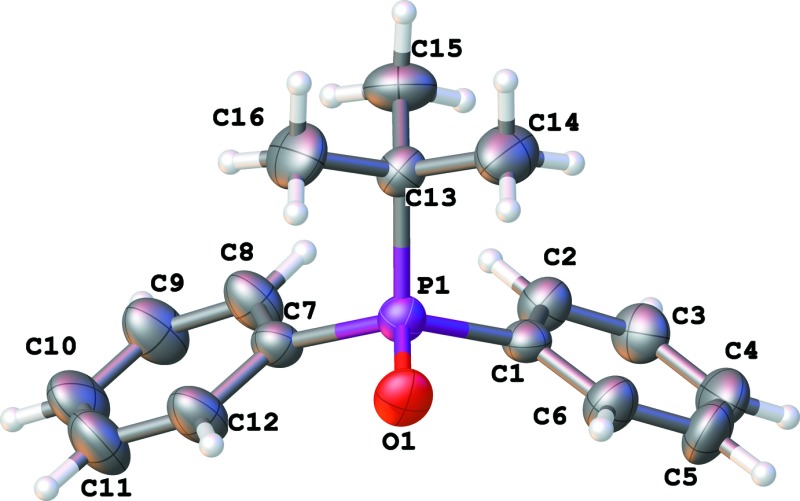
A ball-and-stick representation of the molecular structure of I.

I . DOI: 10.1107/S2056989015008919/hg5439fig1.tif
A ball-and-stick representation of the mol­ecular structure of **I**.

**Fig. 2.**
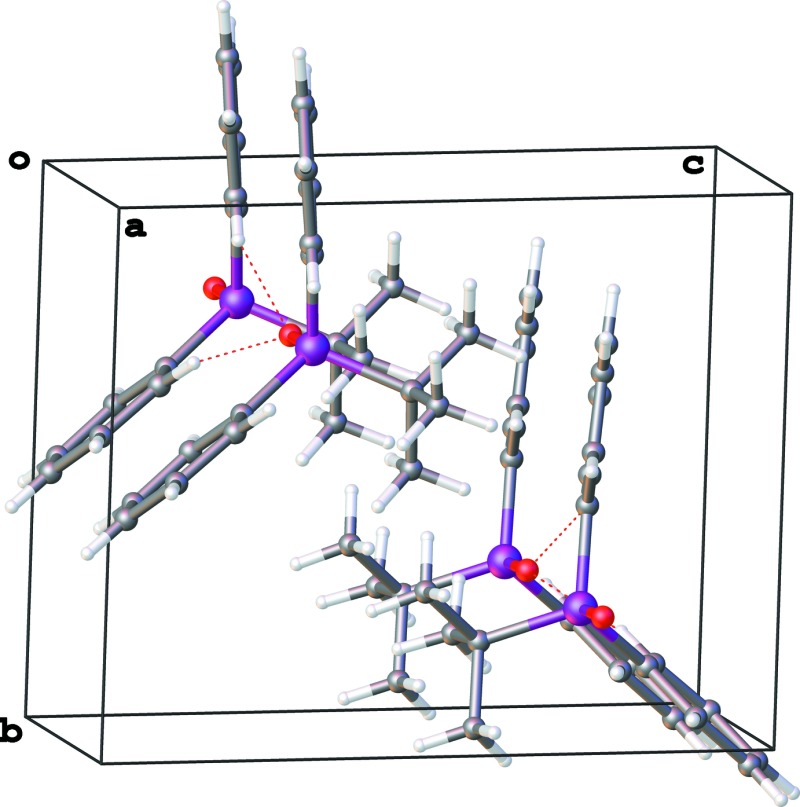
Molecular packing with short C—H···O contacts indicated by dashed lines

. DOI: 10.1107/S2056989015008919/hg5439fig2.tif
Mol­ecular packing with short C—H⋯O contacts indicated by dashed lines

CCDC reference: 1063801

Additional supporting information:  crystallographic information; 3D view; checkCIF report

## Figures and Tables

**Table 1:** Selected bond lengths ()

P1O1	1.4897(14)
P1C1	1.821(3)
P1C7	1.825(3)
P1C13	1.841(3)

## supplementary crystallographic information

### S1. Synthesis and crystallization

The tert-butyl­diphenyl­phosphine oxide was unintentionally obtained during the reaction used to coordinate the unoxidized ligand to gold(I). The product was obtained after mixing tert-butyl­diphenyl­phosphine (0.0600 g, 0.24 mmol) with a solution of (C_4_H_8_S)AuCl (0.0264 g, 0.08 mmol) in tetra­hydro­furan (20 mL) at -80^o^C. The reaction solution was stirred for 3 hours and the solvent removed totally by purging nitro­gen gas into the solution. The residue was then recrystallized from CH_2_Cl_2_/n-hexane mixture within six days. Partial evaporation of the solvent provided quality crystals of the title compound. Yield 90%. Melting point 137^o^C (decomposition). ^1^H NMR (CD_2_Cl_2_) [δ (ppm)]: 1.3(s),7.3(m), 7.6(m), 7.9(m). For ^31^P NMR in CD_2_Cl_2_ [δ (ppm)]: 38.96. Infrared data (cm^-1^): 3032 (C–H, Ar), 2908 (C–H, CH_3_), 1597 (C=C, Ar), 1165 (P=O), 1126 (P–Ar).

### S2. Refinement

Crystal data, data collection and structure refinement details are summarized in Table 1. H-atoms were placed in calculated positions and allowed to ride during subsequent refinement, with *U*_iso_(H) = 1.2*U*_eq_(C) and C—H distances of 0.93 Å for ring hydrogens and with *U*_iso_(H) = 1.5*U*_eq_(O) and C—H distances of 0.96 Å for methyl hydrogens.

### Crystal data

**Table:**

C_16_H_19_OP	*F*(000) = 276
*M**_r_* = 258.28	*D*_x_ = 1.164 Mg m^−^^3^
Monoclinic, *P*2_1_	Mo *K*α radiation, λ = 0.71073 Å
*a* = 6.3432 (6) Å	Cell parameters from 2279 reflections
*b* = 9.5219 (9) Å	θ = 3.3–23.1°
*c* = 12.4556 (15) Å	µ = 0.17 mm^−^^1^
β = 101.665 (10)°	*T* = 293 K
*V* = 736.78 (13) Å^3^	Plate, colourless
*Z* = 2	0.4 × 0.15 × 0.04 mm

### Data collection

**Table:**

Agilent Xcalibur, Eos diffractometer	2713 independent reflections
Radiation source: Enhance (Mo) X-ray Source	2203 reflections with *I* > 2σ(*I*)
Graphite monochromator	*R*_int_ = 0.049
Detector resolution: 16.0514 pixels mm^-1^	θ_max_ = 25.4°, θ_min_ = 2.7°
ω scans	*h* = −7→7
Absorption correction: multi-scan (*CrysAlis PRO*; Agilent, 2014)	*k* = −11→11
*T*_min_ = 0.842, *T*_max_ = 1.000	*l* = −14→14
11029 measured reflections	

### Refinement

**Table:**

Refinement on *F*^2^	Secondary atom site location: difference Fourier map
Least-squares matrix: full	Hydrogen site location: inferred from neighbouring sites
*R*[*F*^2^ > 2σ(*F*^2^)] = 0.044	H-atom parameters constrained
*wR*(*F*^2^) = 0.086	*w* = 1/[σ^2^(*F*_o_^2^) + (0.0343*P*)^2^ + 0.0459*P*] where *P* = (*F*_o_^2^ + 2*F*_c_^2^)/3
*S* = 1.05	(Δ/σ)_max_ < 0.001
2713 reflections	Δρ_max_ = 0.18 e Å^−^^3^
166 parameters	Δρ_min_ = −0.24 e Å^−^^3^
1 restraint	Absolute structure: Flack (1983)
Primary atom site location: heavy-atom method	Absolute structure parameter: 0.19 (10)

### Special details

**Table:**

Geometry. All e.s.d.'s (except the e.s.d. in the dihedral angle between two l.s. planes) are estimated using the full covariance matrix. The cell e.s.d.'s are taken into account individually in the estimation of e.s.d.'s in distances, angles and torsion angles; correlations between e.s.d.'s in cell parameters are only used when they are defined by crystal symmetry. An approximate (isotropic) treatment of cell e.s.d.'s is used for estimating e.s.d.'s involving l.s. planes.
Refinement. Refinement of *F*^2^ against ALL reflections. The weighted *R*-factor *wR* and goodness of fit *S* are based on *F*^2^, conventional *R*-factors *R* are based on *F*, with *F* set to zero for negative *F*^2^. The threshold expression of *F*^2^ > 2σ(*F*^2^) is used only for calculating *R*-factors(gt) *etc*. and is not relevant to the choice of reflections for refinement. *R*-factors based on *F*^2^ are statistically about twice as large as those based on *F*, and *R*- factors based on ALL data will be even larger.

### Fractional atomic coordinates and isotropic or equivalent isotropic displacement parameters (Å^2^)

**Table:**

	*x*	*y*	*z*	*U*_iso_*/*U*_eq_	
P1	0.87488 (9)	0.74951 (7)	0.71978 (5)	0.03371 (18)	
O1	1.1119 (2)	0.7455 (2)	0.72615 (15)	0.0475 (5)	
C2	0.5797 (4)	0.5351 (3)	0.7611 (2)	0.0437 (7)	
H2	0.4792	0.6045	0.7664	0.052*	
C1	0.7771 (4)	0.5727 (3)	0.7363 (2)	0.0349 (7)	
C14	0.7903 (5)	0.7211 (4)	0.4984 (2)	0.0631 (10)	
H14A	0.9440	0.7152	0.5077	0.095*	
H14B	0.7310	0.7586	0.4271	0.095*	
H14C	0.7323	0.6291	0.5050	0.095*	
C6	0.9206 (5)	0.4655 (3)	0.7265 (2)	0.0485 (8)	
H6	1.0527	0.4876	0.7093	0.058*	
C4	0.6771 (5)	0.2920 (3)	0.7685 (3)	0.0565 (9)	
H4	0.6443	0.1988	0.7802	0.068*	
C3	0.5318 (5)	0.3973 (3)	0.7776 (2)	0.0499 (8)	
H3	0.4003	0.3745	0.7951	0.060*	
C7	0.8146 (4)	0.8617 (3)	0.8285 (2)	0.0375 (7)	
C8	0.6180 (4)	0.8754 (3)	0.8609 (3)	0.0513 (8)	
H8	0.4985	0.8257	0.8246	0.062*	
C10	0.7712 (5)	1.0408 (4)	1.0008 (2)	0.0609 (9)	
H10	0.7562	1.0998	1.0583	0.073*	
C9	0.6004 (5)	0.9632 (4)	0.9473 (3)	0.0615 (10)	
H9	0.4692	0.9697	0.9694	0.074*	
C12	0.9867 (4)	0.9407 (3)	0.8838 (3)	0.0543 (9)	
H12	1.1197	0.9334	0.8637	0.065*	
C11	0.9640 (5)	1.0303 (4)	0.9686 (3)	0.0671 (10)	
H11	1.0807	1.0836	1.0036	0.081*	
C5	0.8706 (5)	0.3264 (3)	0.7418 (3)	0.0590 (9)	
H5	0.9682	0.2560	0.7341	0.071*	
C13	0.7324 (4)	0.8175 (3)	0.5864 (2)	0.0361 (6)	
C16	0.8231 (5)	0.9656 (3)	0.5733 (3)	0.0646 (10)	
H16A	0.7806	1.0281	0.6255	0.097*	
H16B	0.7678	0.9994	0.5004	0.097*	
H16C	0.9774	0.9613	0.5858	0.097*	
C15	0.4891 (4)	0.8239 (4)	0.5752 (3)	0.0577 (9)	
H15A	0.4356	0.7323	0.5876	0.087*	
H15B	0.4240	0.8552	0.5028	0.087*	
H15C	0.4543	0.8885	0.6283	0.087*	

### Atomic displacement parameters (Å^2^)

**Table:**

	*U*^11^	*U*^22^	*U*^33^	*U*^12^	*U*^13^	*U*^23^
P1	0.0276 (3)	0.0354 (4)	0.0388 (4)	−0.0001 (4)	0.0084 (3)	0.0003 (4)
O1	0.0296 (8)	0.0516 (11)	0.0634 (12)	0.0015 (12)	0.0142 (8)	−0.0006 (14)
C2	0.0414 (16)	0.0407 (17)	0.0509 (19)	0.0010 (14)	0.0138 (13)	0.0036 (15)
C1	0.0355 (15)	0.0370 (17)	0.0306 (16)	0.0023 (12)	0.0031 (12)	0.0032 (12)
C14	0.081 (2)	0.068 (3)	0.0415 (18)	0.0185 (19)	0.0145 (16)	−0.0007 (17)
C6	0.0436 (17)	0.0429 (19)	0.060 (2)	0.0050 (13)	0.0127 (16)	0.0019 (15)
C4	0.070 (2)	0.036 (2)	0.059 (2)	−0.0047 (15)	0.0044 (17)	0.0081 (14)
C3	0.0496 (18)	0.047 (2)	0.054 (2)	−0.0094 (15)	0.0141 (15)	0.0064 (15)
C7	0.0312 (14)	0.0432 (17)	0.0368 (17)	−0.0002 (13)	0.0036 (12)	−0.0016 (13)
C8	0.0411 (17)	0.067 (2)	0.0468 (19)	−0.0115 (15)	0.0110 (14)	−0.0145 (16)
C10	0.063 (2)	0.078 (2)	0.0397 (19)	0.005 (2)	0.0079 (16)	−0.0177 (19)
C9	0.050 (2)	0.086 (3)	0.052 (2)	−0.0016 (18)	0.0181 (17)	−0.0197 (19)
C12	0.0380 (17)	0.071 (2)	0.052 (2)	−0.0032 (16)	0.0048 (15)	−0.0153 (18)
C11	0.055 (2)	0.081 (3)	0.061 (2)	−0.010 (2)	0.0004 (18)	−0.028 (2)
C5	0.060 (2)	0.0360 (19)	0.081 (3)	0.0131 (16)	0.0143 (18)	−0.0008 (17)
C13	0.0351 (14)	0.0347 (16)	0.0405 (17)	0.0010 (12)	0.0128 (12)	0.0037 (13)
C16	0.078 (2)	0.041 (2)	0.073 (3)	−0.0094 (17)	0.0122 (19)	0.0163 (17)
C15	0.0439 (16)	0.075 (2)	0.051 (2)	0.0054 (16)	0.0026 (15)	0.0143 (18)

### Geometric parameters (Å, º)

**Table:**

P1—O1	1.4897 (14)	C7—C12	1.388 (3)
P1—C1	1.821 (3)	C8—H8	0.9300
P1—C7	1.825 (3)	C8—C9	1.385 (4)
P1—C13	1.841 (3)	C10—H10	0.9300
C2—H2	0.9300	C10—C9	1.368 (4)
C2—C1	1.395 (3)	C10—C11	1.366 (4)
C2—C3	1.372 (4)	C9—H9	0.9300
C1—C6	1.390 (3)	C12—H12	0.9300
C14—H14A	0.9600	C12—C11	1.388 (4)
C14—H14B	0.9600	C11—H11	0.9300
C14—H14C	0.9600	C5—H5	0.9300
C14—C13	1.530 (4)	C13—C16	1.544 (4)
C6—H6	0.9300	C13—C15	1.523 (3)
C6—C5	1.384 (4)	C16—H16A	0.9600
C4—H4	0.9300	C16—H16B	0.9600
C4—C3	1.382 (4)	C16—H16C	0.9600
C4—C5	1.374 (4)	C15—H15A	0.9600
C3—H3	0.9300	C15—H15B	0.9600
C7—C8	1.393 (3)	C15—H15C	0.9600
			
O1—P1—C1	109.48 (12)	C9—C10—H10	120.5
O1—P1—C7	109.63 (12)	C11—C10—H10	120.5
O1—P1—C13	111.29 (10)	C11—C10—C9	119.0 (3)
C1—P1—C7	109.29 (12)	C8—C9—H9	119.2
C1—P1—C13	108.10 (12)	C10—C9—C8	121.6 (3)
C7—P1—C13	109.02 (12)	C10—C9—H9	119.2
C1—C2—H2	119.5	C7—C12—H12	119.4
C3—C2—H2	119.5	C11—C12—C7	121.2 (3)
C3—C2—C1	120.9 (3)	C11—C12—H12	119.4
C2—C1—P1	127.1 (2)	C10—C11—C12	120.4 (3)
C6—C1—P1	115.15 (19)	C10—C11—H11	119.8
C6—C1—C2	117.7 (3)	C12—C11—H11	119.8
H14A—C14—H14B	109.5	C6—C5—H5	119.9
H14A—C14—H14C	109.5	C4—C5—C6	120.1 (3)
H14B—C14—H14C	109.5	C4—C5—H5	119.9
C13—C14—H14A	109.5	C14—C13—P1	106.90 (19)
C13—C14—H14B	109.5	C14—C13—C16	108.9 (2)
C13—C14—H14C	109.5	C16—C13—P1	106.9 (2)
C1—C6—H6	119.4	C15—C13—P1	113.58 (18)
C5—C6—C1	121.2 (3)	C15—C13—C14	110.2 (2)
C5—C6—H6	119.4	C15—C13—C16	110.2 (2)
C3—C4—H4	120.3	C13—C16—H16A	109.5
C5—C4—H4	120.3	C13—C16—H16B	109.5
C5—C4—C3	119.3 (3)	C13—C16—H16C	109.5
C2—C3—C4	120.7 (3)	H16A—C16—H16B	109.5
C2—C3—H3	119.7	H16A—C16—H16C	109.5
C4—C3—H3	119.7	H16B—C16—H16C	109.5
C8—C7—P1	127.2 (2)	C13—C15—H15A	109.5
C12—C7—P1	115.06 (19)	C13—C15—H15B	109.5
C12—C7—C8	117.8 (3)	C13—C15—H15C	109.5
C7—C8—H8	120.0	H15A—C15—H15B	109.5
C9—C8—C7	120.0 (3)	H15A—C15—H15C	109.5
C9—C8—H8	120.0	H15B—C15—H15C	109.5
			
P1—C1—C6—C5	177.2 (3)	C3—C2—C1—C6	1.5 (4)
P1—C7—C8—C9	178.4 (3)	C3—C4—C5—C6	1.3 (5)
P1—C7—C12—C11	−179.9 (3)	C7—P1—C1—C2	43.6 (3)
O1—P1—C1—C2	163.7 (2)	C7—P1—C1—C6	−134.0 (2)
O1—P1—C1—C6	−14.0 (2)	C7—P1—C13—C14	179.33 (18)
O1—P1—C7—C8	−169.2 (3)	C7—P1—C13—C16	62.9 (2)
O1—P1—C7—C12	10.6 (3)	C7—P1—C13—C15	−58.9 (2)
O1—P1—C13—C14	58.3 (2)	C7—C8—C9—C10	1.7 (5)
O1—P1—C13—C16	−58.2 (2)	C7—C12—C11—C10	1.2 (5)
O1—P1—C13—C15	−180.0 (2)	C8—C7—C12—C11	0.0 (5)
C2—C1—C6—C5	−0.6 (4)	C9—C10—C11—C12	−1.0 (5)
C1—P1—C7—C8	−49.2 (3)	C12—C7—C8—C9	−1.4 (4)
C1—P1—C7—C12	130.6 (2)	C11—C10—C9—C8	−0.5 (5)
C1—P1—C13—C14	−62.0 (2)	C5—C4—C3—C2	−0.4 (5)
C1—P1—C13—C16	−178.44 (19)	C13—P1—C1—C2	−75.0 (2)
C1—P1—C13—C15	59.8 (2)	C13—P1—C1—C6	107.4 (2)
C1—C2—C3—C4	−1.0 (4)	C13—P1—C7—C8	68.7 (3)
C1—C6—C5—C4	−0.7 (5)	C13—P1—C7—C12	−111.4 (2)
C3—C2—C1—P1	−176.1 (2)		
