# Supplementary figures and images for: Crystal structure of tert-butyl­diphenyl­phosphine oxide
Source: Acta Crystallogr E Crystallogr Commun. 2015 May 13;71(Pt 6):o400. doi: 10.1107/S2056989015008919 (PMC4459331; doi:10.1107/S2056989015008919)

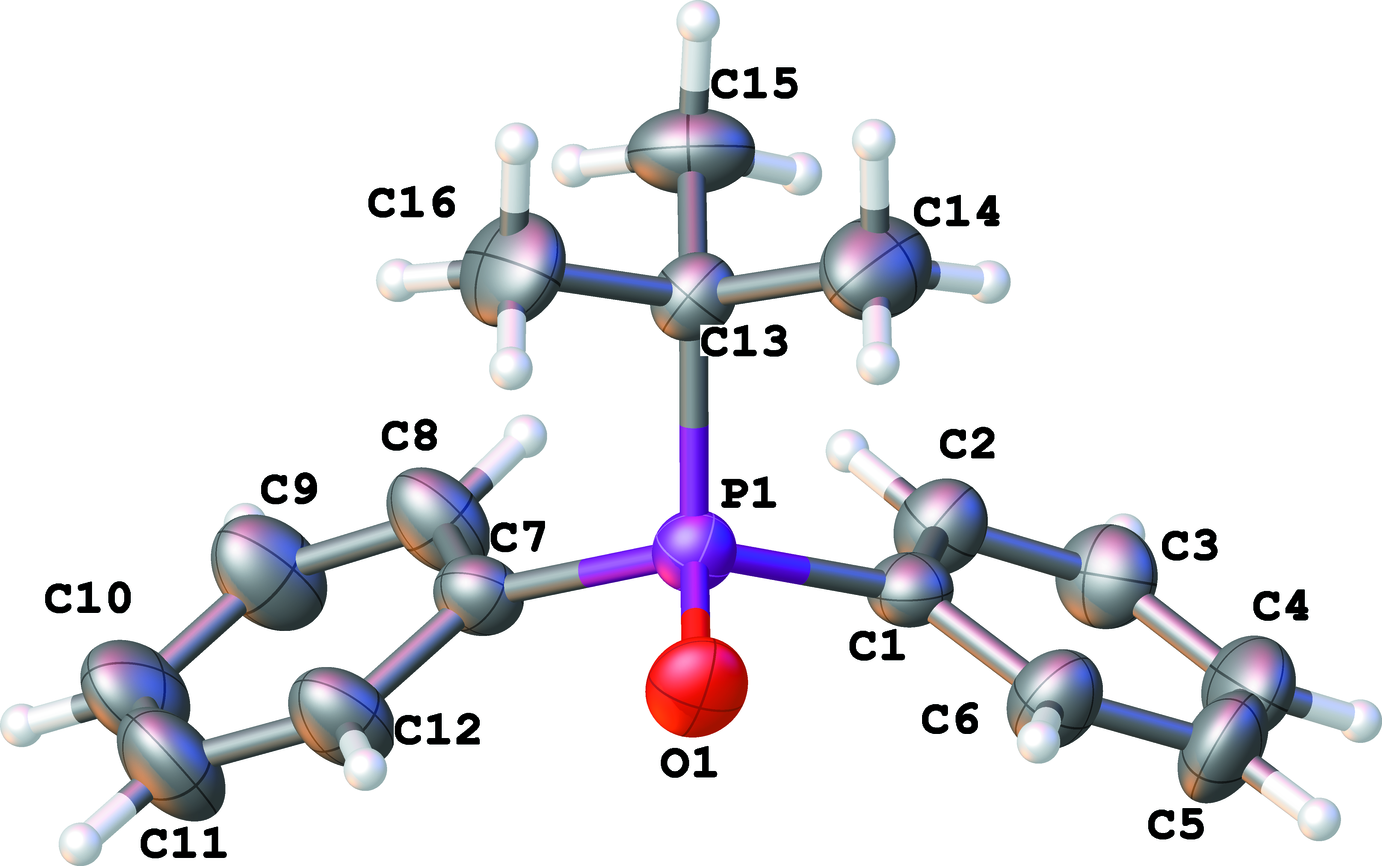

Supplement: Supplementary file 3 [file e-71-0o400-fig1.tif]

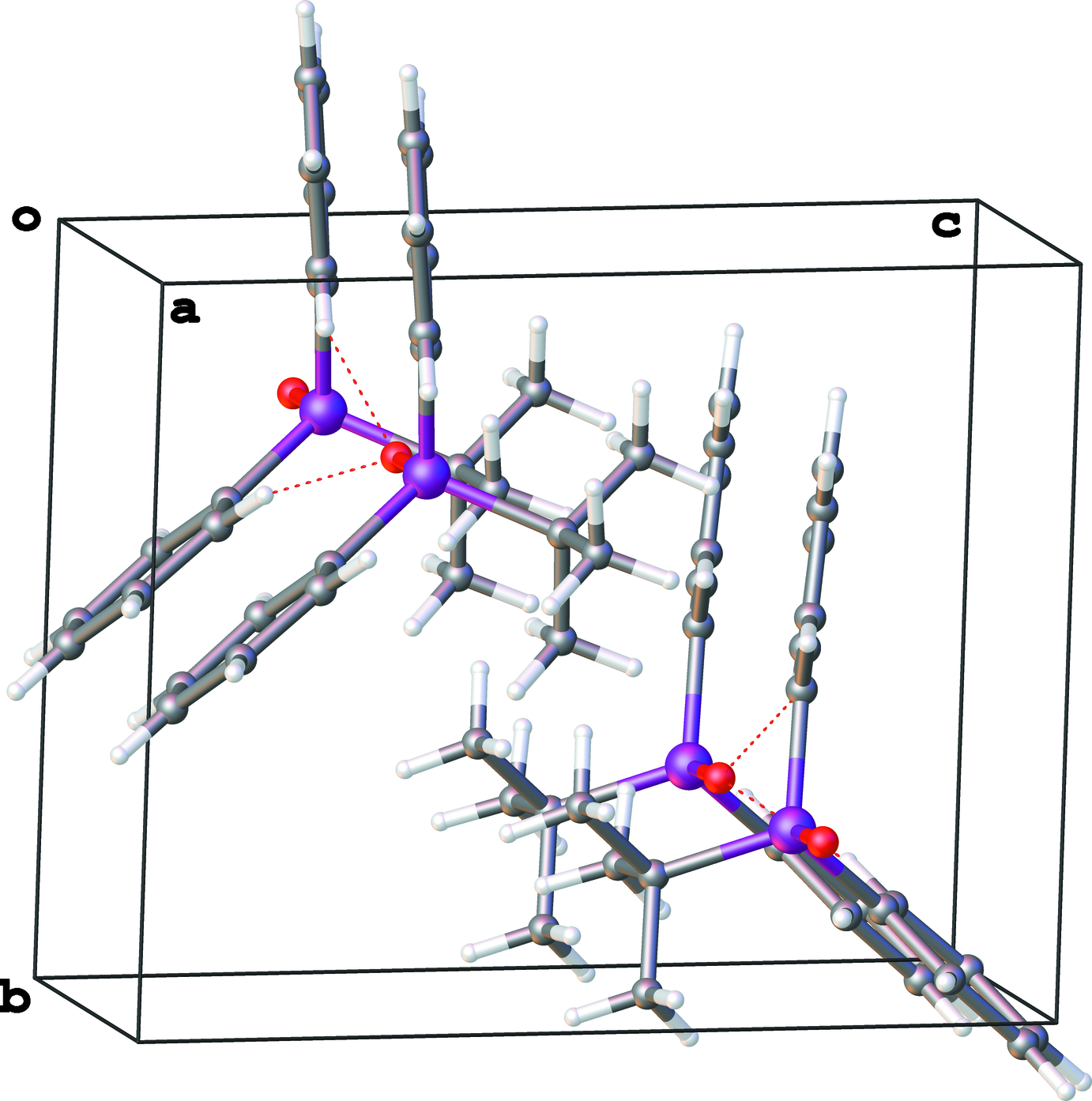

Supplement: Supplementary file 4 [file e-71-0o400-fig2.tif]
